# Evaluation of the SMARTCHEK Genesystem RT-qPCR assay for the detection of SARS-CoV-2 in clinical samples

**DOI:** 10.1186/s12879-022-07319-0

**Published:** 2022-04-04

**Authors:** Diana Flores-León, Willi Quino, Alejandra E. Cúneo, Junior Caro-Castro, Evans Cucho-Meza, Ronnie G. Gavilan

**Affiliations:** 1 Centro Nacional de Salud Pública, Instituto Nacional de Salud, Lima, Peru; 2 Escuela Profesional de Medicina Humana, Universidad Privada San Juan Bautista, Lima, Peru

**Keywords:** COVID-19, SARS-CoV-2, RT-qPCR, Molecular diagnosis

## Abstract

**Background:**

The COVID-19 pandemic remains the main public health problem, due to the quick and easy dissemination of the causal agent, SARS-CoV-2 virus, around the world. Since the beginning of the pandemic, an opportune laboratory diagnosis has been critical to respond this emergency, and RT-qPCR has been used as reference molecular tests for detection of SARS-CoV-2.

**Methods:**

In this study, we performed the evaluation of a RT-qPCR SMARTCHEK platform (SMARTCHEK, Genesystem) for SARS-CoV-2 detection based on the amplification of *RdRp* and *N* gene markers. The platform was evaluated with nasopharyngeal swab samples corresponding to 360 suspected cases of COVID-19 which were remitted to Instituto Nacional de Salud in Peru. This quick method was compared with conventional RT-qPCR as gold standard.

**Results:**

The RT-qPCR SMARTCHEK showed a 98.1% sensitivity (CI: 93.3–99.8%), a 98.8% specificity (CI: 96.6–99.8%), a 97.2% positive predictive value (CI: 92–99.4%) and a 99.2% negative predictive value (CI: 97.2—99.9%). The assay demonstrated a strong agreement between the RT-qPCR SMARTCHEK and conventional RT-qPCR (kappa value ≥ 0.966).

**Conclusion:**

The RT-qPCR SMARTCHEK is a platform that gives reliable and fast results, with high sensitivity and specificity for the detection of SARS-CoV-2, and it will be considered a suitable alternative to COVID-19 diagnosis in low-resource settings.

**Supplementary Information:**

The online version contains supplementary material available at 10.1186/s12879-022-07319-0.

## Background

The Coronavirus disease 2019 (COVID-19) is caused by the Severe Acute Respiratory Syndrome Coronavirus 2 (SARS-CoV-2) which emerged in Wuhan, province of Hubei, China in December, 2019, affecting more than 190 countries all over the world [1, 2]. Until February 15th, 2022, more than 413.7 million cases of COVID-19 have been reported in 191 countries and approximately more than 5.8 million deaths from this disease [3]. Peru is experiencing one of the largest COVID-19 epidemics in Latin America, reporting 3.36 million of confirmed cases and 206 thousand deaths until February 15th, 2022 [4]. Due to the increasing cases of COVID-19, Peruvian government has launched an epidemiological alert to intensify early detection of cases [5].

In most countries, one of the main preventive measures include the rapid diagnosis of COVID-19 cases, thus World Health Organization recommend reverse transcription-PCR real-time (RT-qPCR) as standard test [6–8]. Recently, U.S. Food and Drug Administration (FDA) authorized over 30 in vitro diagnostic methods for SARS-CoV-2 detection under an Emergency Use Authorization (EUA), providing various qPCR alternatives including quantitative reverse transcription-PCR (RT-qPCR), reverse transcription–loop-mediated isothermal amplification (RT-LAMP), and direct rapid RNA extraction-free RT-qPCR [9]. However, most molecular methods present some limitations, including the need of expensive equipment, long periods of processing time, trained laboratory-personal and specialized laboratory infrastructure. Likewise, the access to molecular testing in low-resource communities in Peru is very scarce. For this reason, the developing of faster and precise diagnosis molecular methods applied on in low-resource settings are necessary to improve early detection and control of COVID-19 [10].

To address these limitations, rapid molecular methods as viral RNA detection kit for SARS-CoV-2 (SMARTCHEK, Genesystem) are available, based on a biochip sample format on the amplification of *RdRp* and *N* gene markers which gives around 45-min response time with a simple workflow, offering all the benefits of a RT-qPCR at the same time [11]. Thus, the aim of this study was to evaluate the platform for quick detection of SARS-CoV-2 based on RT-qPCR from clinical samples.

## Methods

### Clinical samples collection

A total of 360 nasopharyngeal swab samples were received by the Instituto Nacional de Salud (INS) for molecular diagnosis by RT-qPCR under the SARS-CoV-2 surveillance system in Peru.

### Viral RNA extraction

The viral RNA of clinical samples was extracted and purified using QIAamp Viral RNA Mini kit (Qiagen, Germany), following the manufacturer recommendations. RNA quantification and quality assessment were evaluated by spectrophotometry using DS-11 FX (DeNovix, USA) and fluorometry using Qubit 3.0 (Invitrogen, USA).

### Conventional RT-qPCR

The conventional reverse transcription-qPCR (RT-qPCR) was performed using the primers and probes described by Corman et al. [7] which are recommended for SARS-CoV-2 detection by the Centers for Disease Control and Prevention (CDC). RT-qPCR amplification of the SARS-CoV-2 which includes *E* (0.4 μM) and *RdRp* (0.8 μM) as protein gene targets were performed using one step RT-qPCR kit Superscript III with Platinum Taq Polymerase (Invitrogen, Germany). The assay uses a human reference gene as amplification control. Cycling conditions were as follows: 15 min at 50 °C, 2 min at 95 °C, and 45 cycles, with 1 cycle consisting of 15 s at 95 °C and 30 s at 58 °C. Rotor Gene Q Qiagen thermocycler was employed for the amplification. To interpret the results, SARS-CoV-2 positive samples was considered when there was an amplification in at least one of two markers with Ct value < 40, and considered negative with Ct values  ≥ 40. In case of internal control (*GAPDH*), positive reaction was considered with Ct value < 40 and negative reaction with Ct values  ≥ 40. In all reactions, the internal control must be positive before the final interpretation.

### RT- PCR SMARTCHEK

The samples were analyzed with RT-qPCR SMARTCHEK (Genesystem, South Korea) detection kit for the novel coronavirus (SARS-CoV-2) following the manufacturer recommendations. The kit is based on a biochip sample format, where primers and probes employed for the detection of *RdRp* and *N* genes specific to SARS-CoV-2 are dehydrated on the chip wells. For each Biochip, 04 patient samples can be detected simultaneously (well 2–9) and an internal control is evaluated in each well. Well number 1 corresponds to the not template control (contains pre-labeled primers and probes) and well number 10 is assigned to the positive control (contains pre-labeled primers and probes along with positive templates) (Additional file 1: Fig S1).

For the preparation of the sample reaction for RT-qPCR, a mix of 10 μL of Premix and 10 μL of viral RNA was used. The mix was inoculated in duplicate on the chip; each single assay allows detecting *N* and *RdRp* genes, respectively. Each Biochip can detect 4 patient samples simultaneously for two markers, including internal and negative control.

The RT-qPCR assay was performed in the Genechecker UF-300 (Genesytem, South Korea) platform, using temperature condition including 10 min at 50 °C, 30 s at 95 °C, and 40 cycles, with 1 cycle consisting of 5 s at 95 °C, 20 s at 58 °C and 5 s at 72 °C. The presence of SARS-CoV-2 virus was detected by the identification of an amplification curve with Ct ≤ 37 value for *RdRp* gene, *N* gene and the amplification of internal control. In all reactions, the internal control must be positive to allow for results to be interpreted.

### Analytical sensitivity of the RT-qPCR SMARTCHEK

The limit of detection (LOD) of RT-qPCR and RT-qPCR SMARTCHEK were compared. LoD was calculated using the positive control 2019-nCoV_RdRp (*ORF1ab*) (Integrated DNA Technologies), which contains the envelope gene and a portion of the RNA-dependent RNA polymerase (*RdRp*) and the positive control 2019-nCoV_E (Integrated DNA Technologies), both controls have been synthesized at a concentration of 200,000 copies/μL. Predetermined copy numbers of biochemically synthesized RNA were serially diluted ten-fold from 10^6^ copies to 10^–1^ copies of the target gene per reaction. The linearity test of both methodologies was evaluated by means of linear regression analysis (average of the Ct values versus logarithmic concentration values).

### Analytical Specificity of RT-qPCR SMARTCHEK

Cross-reactivity of SMARTCHEK RT-qPCR was evaluated from fourteen viral RNAs: influenza viruses (A and B), respiratory syncytial virus, human metapneumovirus, rhinovirus, Zika virus, and Human Immunodeficiency Virus (HIV).

### Evaluation of RT-qPCR in clinical samples

In the evaluation of the diagnostic sensitivity and specificity of RT-qPCR SMARTCHEK, the primers and probes described by Corman et al. [7] were used, taking the conventional RT-qPCR as reference method. A total of 360 nasopharyngeal swab samples were used for the study. An estimation of diagnostic sensitivity, specificity, positive predictive value and negative predictive value of RT-qPCR SMARTCHEK was performed.

### Statistical analysis

The analysis of the diagnostic accuracy data was performed with the statistical program Stata v16.0 (Stata Corporation, College Station, TX, USA). Sensitivity, specificity, positive predictive value (PPV), and negative predictive value (NPV) of SMARTCHEK RT-qPCR were estimated. Likewise, the results obtained between both methodologies were compared. All point estimators obtained were accompanied by their 95% confidence interval (95% CI).

To compare the linearity of the RT-qPCR SMARTCHEK assay and the RT-qPCR, the concentrations of the serial dilutions were plotted on the X axis and the average Ct of the three replicates performed on the Y axis. Correlation coefficient R^2^ obtained in the linear regression was used to determine the linearity of the RT-qPCR assay. LoD was calculated by Probit regression analysis, at 95% probability to detect the analytic limit that can be reliably detected by molecular assays, using MedCalc v19.21 software. The degree of agreement was quantified by Cohen’s kappa statistic and correlation between methodologies was evaluated using the Spearman’s correlation coefficient. Also, Ct values were evaluated using the intraclass correlation coefficient and Bland–Altman analysis. On the Y axis, the differences in Ct between both methods (AB) were represented, while on the X axis, the average of the two measurements was represented (A + B/two).

## Results

### Analytical sensitivity of RT-qPCR SMARTCHEK

**Fig. 1:**
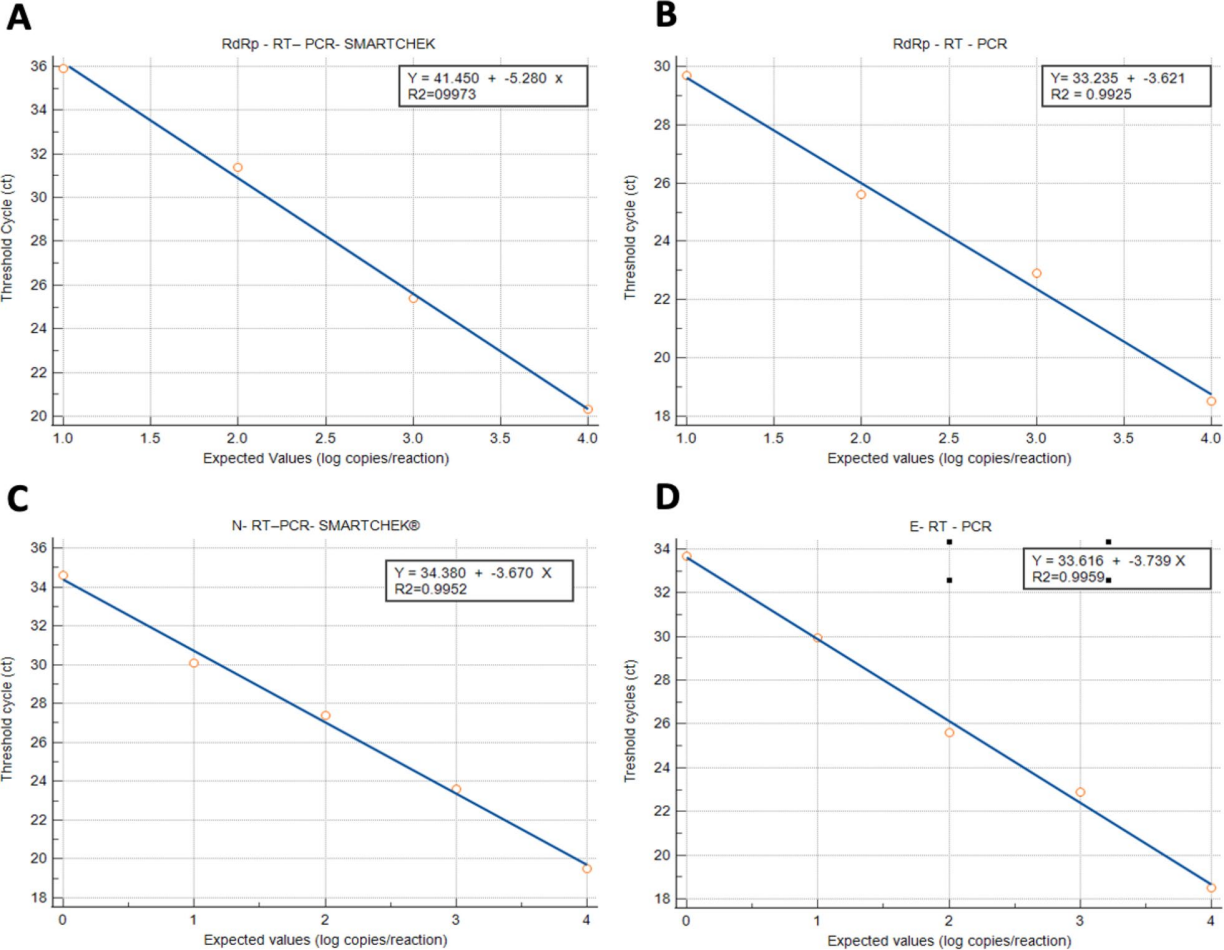
Graph of the linearity assay to determine the LoD of the RT-qPCR and the RT-qPCR SMARTCHEK. **A**, **B** (Control 2019-nCoV_RdRp (ORF1ab)): The expected values (converted to log_10_) were plotted on the X axis, and the average of the RT-qPCR Ct obtained from the three replicates was plotted on the Y axis (A is for *RdRp* gene evaluated by RT-qPCR SMARTCHEK and B is for *RdRp* gene evaluated by RT-qPCR). **C**, **D** ( Control 2019-nCoV_E): Expected values (converted to log_10_) were plotted on the X axis, and the average of the RT-qPCR Ct obtained from the three replicates were plotted on the Y axis (C is for *N* gene and D is for *E* gene). Data are representative of three independent experiments with 3 replicates for each concentration. The software used was MedCalc v19.2.1

Detection limits were compared by RT-qPCR and RT-qPCR SMARTCHEK for the*RdRp* gene, obtaining a LoD of 20 copies/reaction and 200 copies/reaction with R^2^ = 0.9925 and 0.9973, respectively (Fig. 1A, B). In the same way, the *N* gene and the* E* gene were evaluated (RT-qPCR SMARTCHEK), both obtained a LoD of 20 copies/reaction with an R^2^ = 0.9952 and 0.9959 (Fig. 1C, D), respectively. The results showed a lower detection range of RT-qPCR SMARTCHEK compared to RT-QPCR for the *RdRp* gene. A minimum range of difference between *N* and *E* gene detection was also evidenced by RT-qPCR SMARTCHEK and RT-qPCR.

**Fig. 2:**
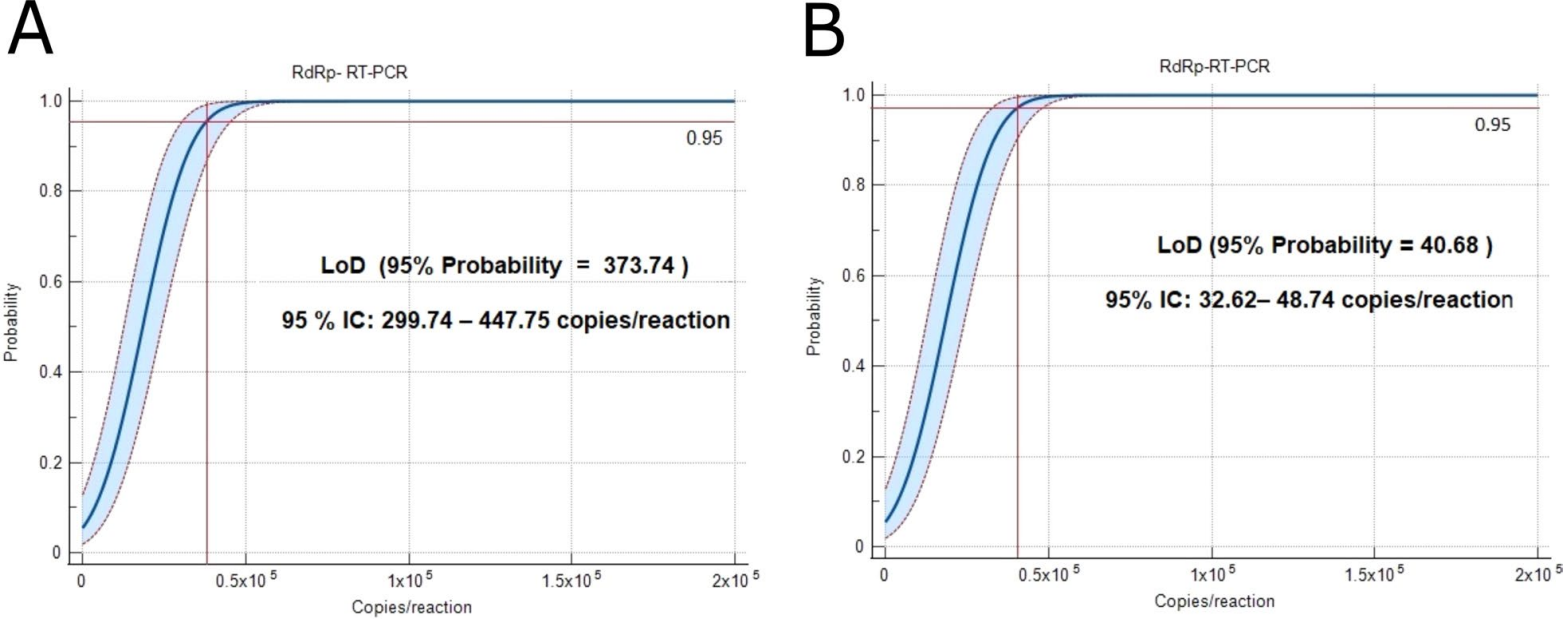
Probit analysis of the sigmoid curve that informs the LoD of RT-qPCR SMARTCHEK and RT-qPCR. **A** *RdRP* gene for RT-qPCR SMARTCHEK and (**B**) *RdRP* gene for RT–qPCR. The X axis shows the expected concentration (copies/reaction). The Y-axis shows a fraction of positive results in all parallel reactions performed. The inner line is a probit curve. Outer lines are the 95% confidence interval (95% CI). The data is representative of three independent experiments with 8 replicates for each concentration

In Fig. 2, the LoD (95% probability) of the *RdRp* gene was identified, being 373.74 (95% CI: 299.74–447.75) copies/reaction using RT-qPCR SMARTCHEK, and 40.68 (95% CI: 32.62–48.74) copies/reaction using RT-qPCR (Fig. 2A, B). In the case of *E* gene by RT-qPCR and *N* gene by RT-qPCR SMARTCHEK, they also presented a LoD of 40.68 (95% CI: 32.62–48.74) copies/reaction. The detection of SARS-CoV-2 at a probability of 95% presents a very similar detection limit for both methodologies.

### Analytical specificity of RT-qPCR SMARTCHEK

From the evaluation of the analytical specificity of the RT-qPCR SMARTCHEK, it has been shown that the fourteen viral RNAs: influenza viruses (A and B), respiratory syncytial virus, human metapneumovirus, rhinovirus, Zika virus and HIV were not detected by the methodology evaluated (Table 1).

**Table 1:** Evaluation of RT-qPCR cross-reaction with other viruses for the *RdRp* and *N* genes detection

Viruses	*RdRp*		*N*		Result
	FAM	ROX	FAM	ROX	
Influenza A—06	NoD	24.45	NoD	23.45	Negative
Influenza A—07	NoD	23.45	NoD	24.76	Negative
Influenza B—08	NoD	24.56	NoD	2456	Negative
Influenza A—12	NoD	24.67	NoD	23.56	Negative
Influenza A—20	NoD	26.37	NoD	24.99	Negative
Zika virus—7	NoD	27.02	NoD	26.06	Negative
HIV—357	NoD	27.9	NoD	25.28	Negative
HIV	NoD	26.72	NoD	25.29	Negative
Zika virus	NoD	28.4	NoD	25.81	Negative
Metapneumovirus	NoD	28.21	NoD	24.98	Negative
Influenza A-05	NoD	27.21	NoD	24.26	Negative
Respiratory Syncytial Virus	NoD	28.68	NoD	24.86	Negative
Rhinovirus	NoD	27.88	NoD	23.4	Negative
Influenza A-04	NoD	26.25	NoD	24.72	Negative

*NoD* non detected

### Diagnostic accuracy of RT-qPCR SMARTCHEK and RT-qPCR

The conventional RT-qPCR based on the *RdRp* and *E* genes detected a total of 100 positives and 260 negatives. This method was used as gold standard on the evaluation of the RT-qPCR SMARTCHEK based on the *RdRp* and *N* genes amplification, which detected 105 positives and 255 negatives; the quick RT-qPCR showed 98.1% sensitivity (CI: 93.3–99.8%), 98.8% specificity (CI: 96.6–99.8%), 97.2% positive predictive value (CI: 92–99.4%) and 99.2% negative predictive value (CI: 97.2–99.9%) (Table 2). The RT-qPCR SMARTCHEK is a platform that gives reliable and fast results, with high sensitivity and specificity for the detection of SARS-CoV-2.

**Table 2:** Estimators of the RT-qPCR SMARTCHEK

Estimator	Value	95% CI^a^
Prevalence	29%	25–34.2%
Sensitivity	98.1%	93.3–99.8%
Specificity	98.8%	96.6–99.8%
Positive predictive value	97.2%	92–99.4%
Negative predictive value	99.2%	97.2–99.9%
Area under ROC curve	0.985	0.97–0.999
Likehood positive ratio	83.4	27.1–257
Likehood negative ratio	0.0193	0.0049–0.0761

^a^*95%CI* 95% confidence interval

### Comparative analysis of discordant results between SMARTCHECK RT-qPCR and conventional RT-qPCR

**Fig. 3:**
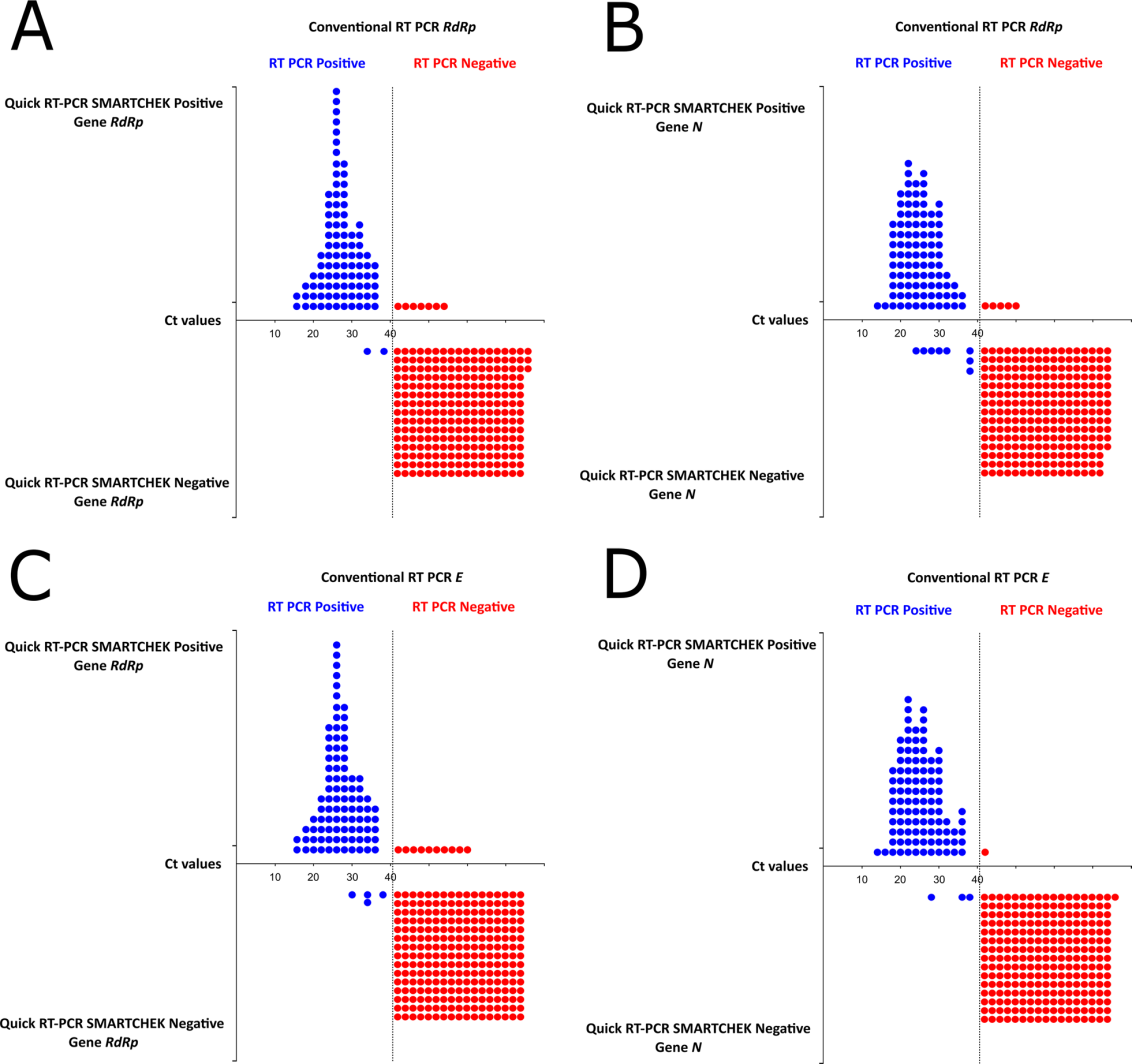
Linearity chart comparing the RT-qPCR SMARTCHEK *RdRp/N* positive/negative samples and their detection based on the *RdRp/E* gene-based conventional RT-qPCR. **A** RT-qPCR SMARTCHEK *RdRp* positive/negative vs *RdRp* gene-based conventional RT-qPCR. **B** RT-qPCR SMARTCHEK *N* positive/negative vs *RdRp* gene-based conventional RT-qPCR. **C** RT-qPCR SMARTCHEK positive/negative vs *E* gene-based RT-qPCR. **D** RT-qPCR SMARTCHEK *N* positive/negative vs *E* gene-based conventional RT-qPCR

In the comparative analysis of the *RdRp* gene by both methodologies, 7 false negatives and 2 false positives samples were observed (Fig. 3A). The *N* gene of the RT-qPCR SMARTCHEK was also compared with the *RdRp* gene of the RT-qPCR, obtaining a lower number of false positives (n = 8) and a higher number of false negatives (n = 5) (Fig. 3B). The *RdRp* gene of the RT-qPCR SMARTCHEK was also compared with the *E* gene of the RT-qPCR, identifying 10 false negatives and 4 false positives (Fig. 3C), likewise the *N* and *E* genes were compared, showing a lower number of false positives (n = 3) (Fig. 3D).

**Fig. 4:**
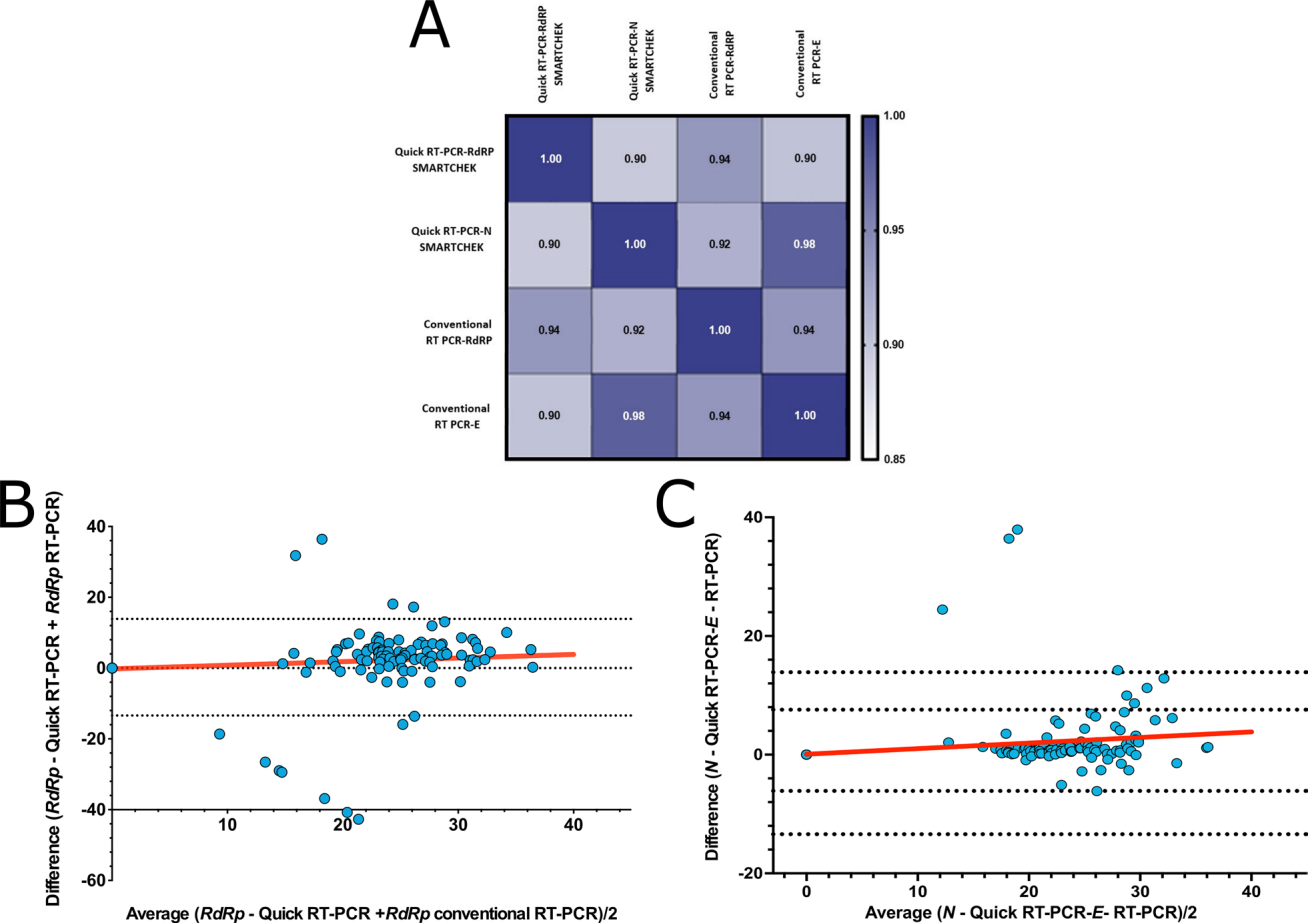
Concordance and statistical relationship of RT-qPCR SMARTCHEK and conventional RT-qPCR. **A** Correlation coefficient between *RdRp*-RT-qPCR SMARTCHEK, *N*-RT-qPCR SMARTCHEK, *RdRp*-conventional RT-qPCR and *E*-conventional RT-qPCR. **B** Bland–Altman graphic showing the difference versus average to compare the Ct value of *RdRp*-RT-qPCR SMARTCHEK and *RdRp*-conventional RT-qPCR. **C** Bland–Altman graphic showing the difference versus average to compare the Ct value of *N*-RT-qPCR SMARTCHEK and *E*-conventional RT-qPCR

The concordance between the *RdRp* and *N* gene-based RT-qPCR SMARTCHEK and conventional RT-qPCR which targeted the *RdRp* and *E* gene were further evaluated through the Cohen’s kappa index, obtaining a value of 0.966 (CI: 95%: 0.937–0.996) (p < 0.001), indicating a good statistic concordance between the evaluated diagnostic tests. Also, the correlation of the individual tests using the Spearman’s correlation between *RdRp* gene-based RT-qPCR SMARTCHEK, *N* gene-based RT-qPCR SMARTCHEK, *RdRp* gen-based conventional RT-qPCR and *E* gene-based conventional RT-qPCR showed a high correlation between them (CI: 0.93 and 0.97 (Fig. 4A).

Using the Bland–Altman representation, the Ct values were evaluated by comparing the results obtained between the *N* marker of the RT-qPCR SMARTCHEK and the *E* marker of the RT-qPCR; in the same way, the *RdRp* marker was compared between both methodologies (Fig. 4B, C). Despite the presence of outliers, a similar sensitivity was obtained between the RT-qPCR SMARTCHEK and the RT-qPCR.

## Discussion

COVID-19 pandemic has demonstrated being an important public health problem for its quick and easy dissemination in the world, showing the importance to count with reliable diagnostic methods for the detection of the SARS-CoV-2 virus. Currently, a lot of investigation institutions around the world are focusing on implement new detection technologies based on RT-qPCR, in order to contribute with the opportune and fast diagnose of patient with suspected of COVID-19 [12].

Since the pandemic started, an important number of prevention measures have been proposed, which include providing an opportune diagnosis [6, 8, 13]. The RT-qPCR test is recommended by the World Health Organization (WHO) as a standard test [6] and is used as a reference for the validation of alternative molecular test for diagnosis such as the validation of commercial kits with clinical samples. In this context, it is necessary to implement new alternatives of molecular tests to RT-qPCR that are low cost, easy to use, and that can bring quick results with a high sensitivity and specificity, in order to strengthen prevention in areas where the access to molecular test is limited.

During the pandemic, different specific primers and probes for the SARS-CoV-2 detection had been accomplished, showing that the *ORF1ab* gene, in spite of being conserved, is less sensitive than other target genes [14]. The RT-qPCR SMARTCHEK has been raised for the detection of the RNA-dependent RNA polymerase (*RdRp*) which encodes a fragment of the *ORF1b* region and shows a high level of intragroup conservation; therefore, it is an ideal target for its application on the diagnosis [15, 16]. Also, the RT-qPCR detects the *N* gene, which is less conserved but more sensitive than other target genes [7]. In the current validation, it has been shown that the *RdRp* gene is more sensitive and specific than the *N* gene using as gold standard the RT-qPCR recommended by the WHO [6, 8]. The RT-qPCR SMARTCHEK provides a mixed result of both genes achieving a sensitivity of 98.1% and a specificity of 98.8%, highly comparable by the methodology proposed by Corman et al. who obtained a sensitivity of 95% and a specificity of 100% [7]. In other investigations, it had also been proved that diagnostic tests enhance the efficacy of the detection [17, 18].Despite having sensitivity and specificity values above 98%, false positives and negatives have been detected oriented to samples with a low viral load of SARS-CoV-2 with very low Ct values; these results also tend to appear in other methodologies for detecting the SARS-CoV-2 virus [23, 24], being the main inconvenience transporting the sample to the processing laboratories.

The LoD of the RT-qPCR SMARTCHEK using the synthetic control 2019-nCoV_RdRP (ORF1ab) presented a lower logarithmic unit than the RT-qPCR *RdRp* gene proposed by Corman et al. [24]; and the synthetic control 2019-nCoV_E presented the same LoD as the RT-qPCR; these data are very similar to those reported by Chang et al. [15] using an in vitro viral RNA, obtaining a LoD of 11.2 copies/reaction of RNA and 21.3 copies/reaction of RNA on the COVID-19-*RdRp* and COVID-19-*N*, respectively. Also, RT-qPCR SMARTCHEK was sensitive enough by detecting the SARS-CoV-2 in a clinical sample, obtaining a LoD for the *RdRp* and *N* genes until 10^4^dilution of RNA in the sample, similar to the description made by Zou et al. [20]; Pan et al. [21] and Wölfel et al. [22], who have shown that infected people had in their majority a high viral charge (between 10^4^ and 10^8^ copies of genome/mL for nasopharyngeal or saliva sample) during the first day of initial symptoms and probably during the pre-syndromic phase.

Finally, this study has some limitations including the necessity to perform RNA extraction before PCR reaction and the limited volume of clinical samples to perform the parallel control. Despite these limitations, RT-qPCR SMARTCHECK is a cost-effective method based on portability that provides a rapid RT-qPCR reaction and a comparable specificity/sensitivity with standard RT-qPCR.

## Conclusion

In conclusion, the RT-qPCR SMARTCHEK could be an alternative to give an opportune diagnosis of SARS-CoV-2, being a platform that gives fast results, with high sensitivity and specificity, having the possibility to improve it by using a quick extraction method that can be used in laboratories of the primary health-care attention from countries which do not have an appropriate structure or adequate equipment.

## Supplementary Information

**Additional file 1: Fig. S1. **Workflow of RT-qPCR SMARTCHECK.

## Data Availability

All data generated or analysed during this study are included in this published article.

## Abbreviations

CDC: Center for Disease control and prevention
CI: Confidence interval
COVID-19: Coronavirus disease 2019
Ct: Cycle threshold
EUA: Emergency use authorization
FDA: Food and Drug Administration
FN: False negatives
FP: False positives
HIV: Human immunodeficiency virus
INS: Instituto Nacional de Salud
LoD: Limit of detection
NoD: Non-detected
RNA: Ribonucleic acid
RT-LAMP: Reverse transcription-loop-mediated isothermal amplification
RT-qPCR: Reverse transcription-qPCR real-time
SARS-CoV-2: Severe acute respiratory syndrome
TN: True negatives
TP: True positives
WHO: World Health Organization
