# Supplementary material for: Evaluation of the SMARTCHEK Genesystem RT-qPCR assay for the detection of SARS-CoV-2 in clinical samples
Source: BMC Infect Dis. 2022 Apr 4;22:329. doi: 10.1186/s12879-022-07319-0 (PMC8977556; doi:10.1186/s12879-022-07319-0)
Supplement: Supplementary file 1 — Additional file 1: Fig. S1. Workflow of RT-qPCR SMARTCHECK. [file 12879_2022_7319_MOESM1_ESM.pdf]

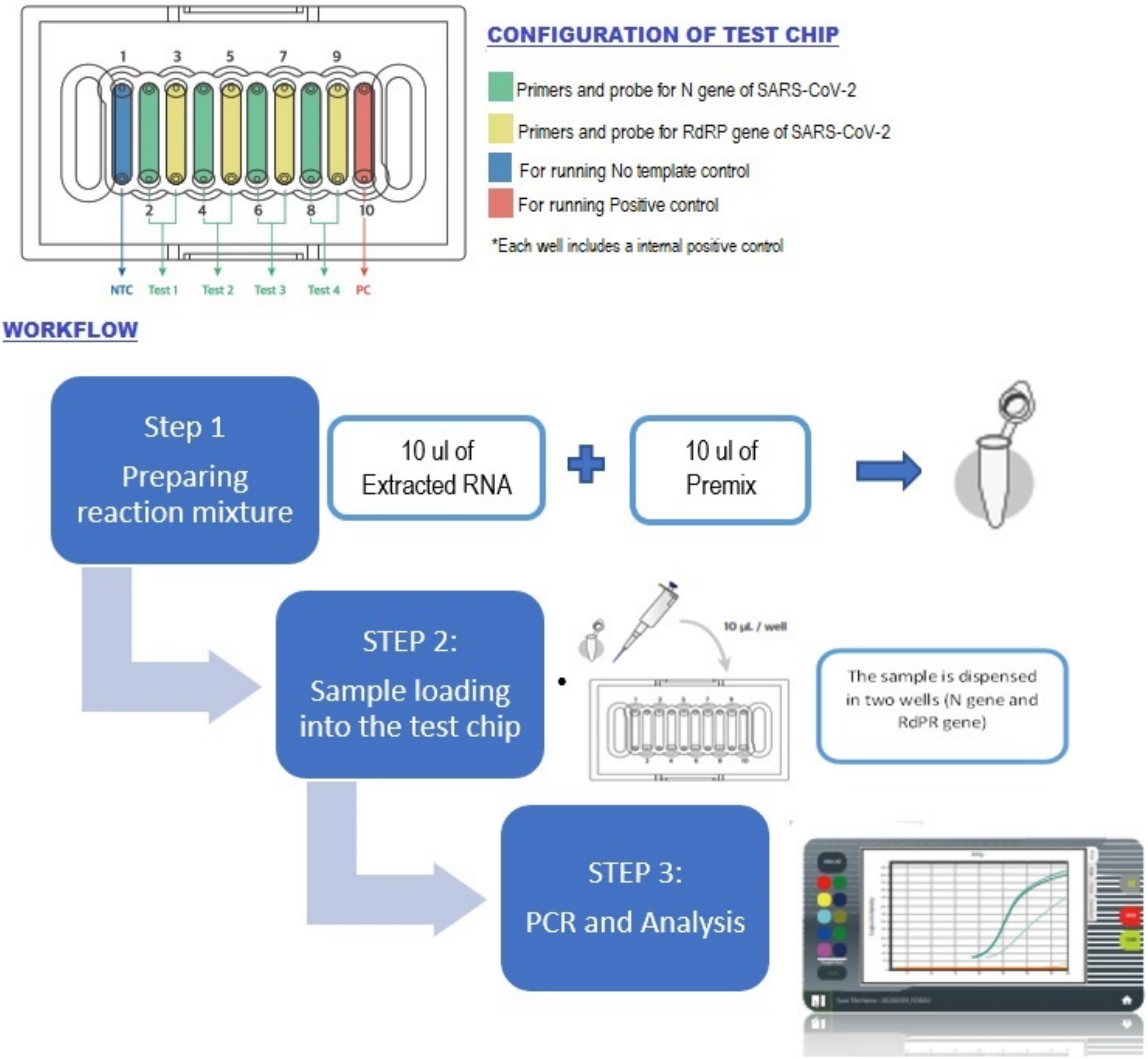

Fig. S1. Workflow of RT-qPCR SMARTCHECK
